# Scalp infestation by Pthirus pubis in a 91-year-old woman: A rare cause of chronic scalp pruritus

**DOI:** 10.1016/j.idcr.2026.e02788

**Published:** 2026-09-28

**Authors:** Floriane Etheve, Estelle Perraud, Damien Boutin

**Affiliations:** a Department of Dermatology, Centre Hospitalier Universitaire de Poitiers, 2 rue de la Milétrie, Poitiers, 86000, France; b Department of Parasitology and Medical Mycology, Centre Hospitalier Universitaire de Poitiers, 2 rue de la Milétrie, Poitiers, 86000, France

**Keywords:** Elderly patient, Pediculosis pubis, Phthiriasis, *Pthirus pubis*, Scalp infestation, Scalp pruritus

## Abstract

*Pthirus pubis* is an ectoparasite that typically infests pubic hair and is primarily transmitted through close sexual contact. Although extragenital involvement may occur, scalp infestation remains exceptionally uncommon. We report the case of a 91-year-old woman who presented with a two-month history of persistent scalp pruritus that failed to respond to oral terbinafine and ketoconazole shampoo. Dermatological examination revealed numerous adult lice and nits attached to scalp hairs, with additional involvement of the eyelashes, eyebrows, and pubic region. Microscopic examination confirmed *Pthirus pubis* infestation. Screening for sexually transmitted infections was negative. Treatment with topical permethrin combined with oral ivermectin led to complete clinical and parasitological resolution. This case highlights the importance of considering *Pthirus pubis* in the differential diagnosis of chronic scalp pruritus, even in very elderly patients. Careful examination of all hair-bearing areas is essential to establish an early diagnosis and avoid inappropriate treatment.

## Introduction

*Pthirus pubis* is an obligate human ectoparasite responsible for pediculosis pubis, a condition generally regarded as a sexually transmitted infestation affecting terminal hairs of the pubic region [1]. Extragenital localizations, including the eyelashes, eyebrows, axillae, beard, and chest, have occasionally been reported [2,3]. In contrast, scalp involvement remains exceptional, with only a limited number of cases reported in the literature, because the morphology of *P. pubis* is adapted to grasp coarse terminal hairs rather than scalp hair [[4], [5], [6], [7], [8], [9]].

Because clinicians rarely suspect *P. pubis* infestation in elderly patients presenting with isolated scalp pruritus, diagnosis may be considerably delayed. This report highlights an unusual presentation and reviews previously published cases.

## Case presentation

A 91-year-old woman with a medical history of insulin-treated diabetes mellitus, hypertension, dyslipidemia, previous hysterectomy with bilateral oophorectomy, and basal cell carcinoma was referred for persistent scalp pruritus that had been evolving for two months.

Before referral, she had received oral terbinafine (250 mg daily) and ketoconazole shampoo without clinical improvement because a fungal scalp disorder had initially been suspected.

Dermatological examination revealed numerous excoriations associated with multiple adult lice and viable nits firmly attached to scalp hairs (Fig. 1).

**Fig. 1:**
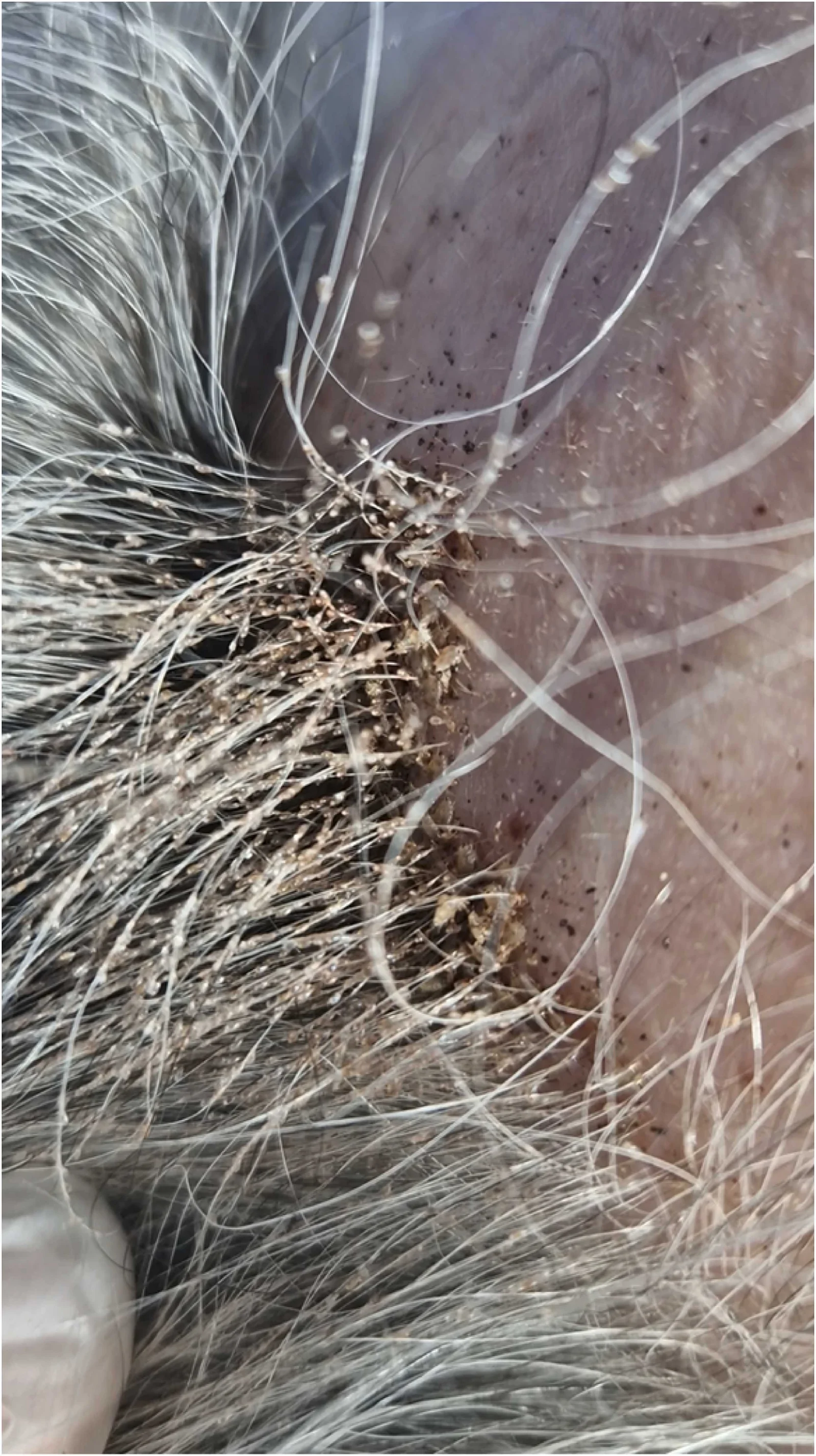
Dermatological examination of the scalp showing numerous adult lice and nits firmly attached to scalp hairs.

Further examination disclosed similar infestation of the eyelashes, eyebrows, and pubic hair, whereas no axillary involvement was observed.

Infested hairs bearing lice and nits were manually collected from the scalp and sent to the parasitology laboratory for microscopic examination. Microscopic examination confirmed the diagnosis of Pthirus pubis infestation. Only adults were observed, measuring 1–2 mm. They are dorsoventrally flattened, typically broader and shorter than head and body lice. They have two antennae and three pairs of legs. The first pair is smaller and thinner than the other two pairs. The second and third pairs of legs end in grasping claws, allowing them to grip the hair tightly. On the abdomen, four pairs of tubercles are covered with setae (Fig. 2).

**Fig. 2:**
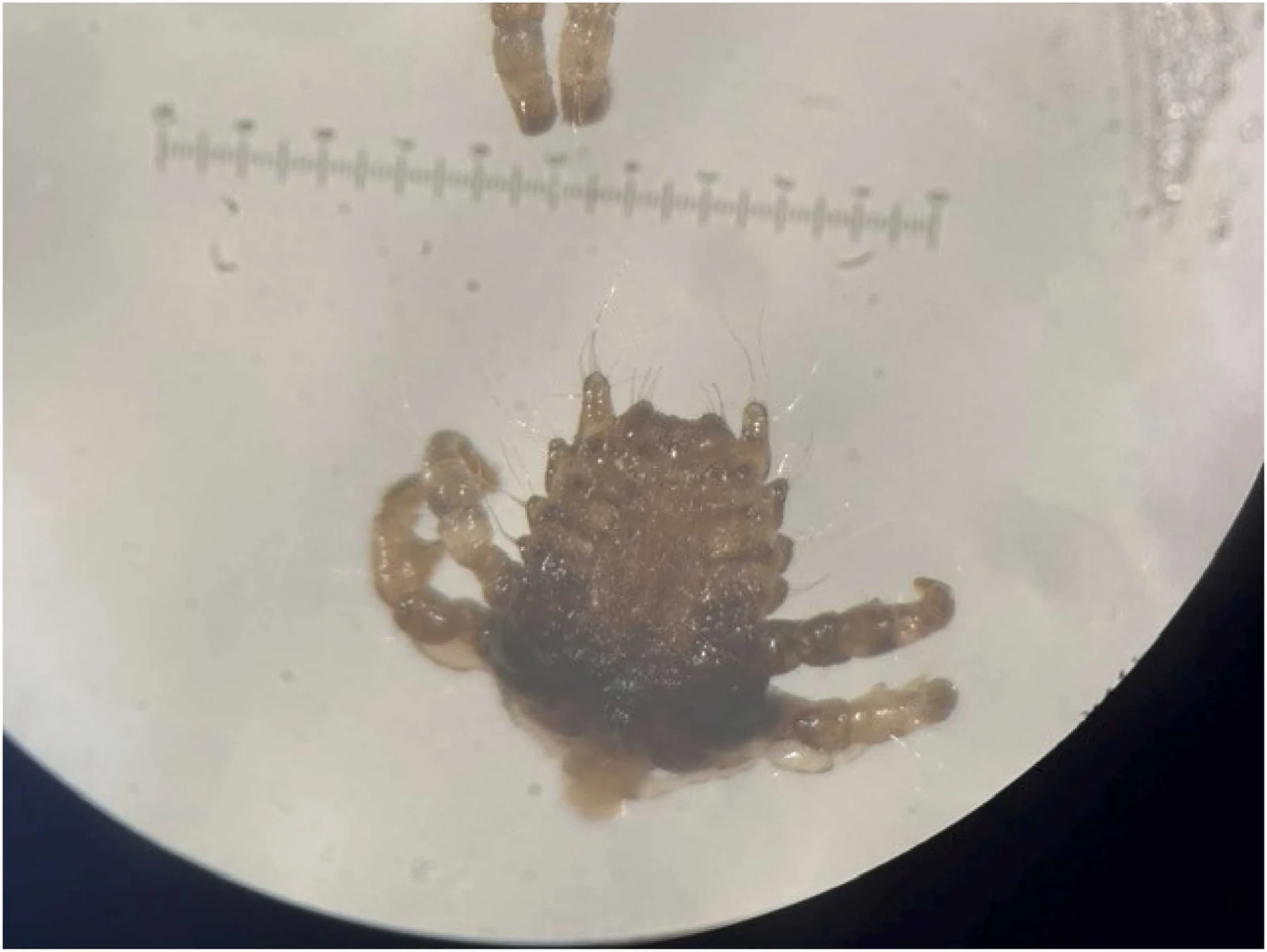
Microscopic examination confirming the diagnosis of Pthirus pubis infestation, showing characteristic adult crab lice and viable nits (×100 magnification).

Routine laboratory investigations were unremarkable. Serological screening for *human immunodeficiency virus* (HIV), hepatitis B virus, hepatitis C virus, and syphilis was negative. No similar symptoms were identified among close contacts. The patient had no significant cognitive impairment and lived with her adult son, who was examined and showed no evidence of infestation. No sexual exposure or other identifiable source of transmission was identified.

Treatment consisted of topical permethrin 1% applied to all affected hair-bearing areas together with oral ivermectin (200 µg/kg) administered on days 1 and 8. At the end of treatment, clinical reassessment showed complete resolution of the infestation, with no live lice or nits detected. No further in-person clinical follow-up was performed. However, during a telephone follow-up approximately 15 days later, the patient reported no recurrence of symptoms or infestation.

## Discussion

Pediculosis pubis most commonly affects sexually active adults and is usually confined to the pubic region [1]. Extragenital localizations are uncommon, with eyelash infestation representing the best-recognized atypical presentation [2,10]. However, P. pubis may involve other hair-bearing sites, including unusual locations such as the ear, as recently reported by Veraldi et al. [11]. In contrast, scalp involvement by *Pthirus pubis* has only rarely been described [4,[6], [7], [8], [9],^12^].

The near-exclusive occurrence of scalp phthiriasis in infants and young children in the published literature reflects the biology of the parasite. The tarsal claws of *P. pubis* are morphologically adapted to grasp short, thick, and widely spaced terminal hairs, of a diameter and interfollicular spacing comparable to pubic, axillary, or eyelash hair, rather than the finer, more densely packed hairs of the scalp [4,13]. Before puberty, pubic and axillary hair has not yet developed. The scalp may therefore represent the most suitable available habitat for *P. pubis*, which may explain why the majority of reported scalp infestations occur in this age group [4,8,13].

Unlike in children, no established biological explanation exists for scalp infestation in elderly individuals. Rather than reflecting a specific age-related tropism, scalp involvement may represent secondary extension of a longstanding multifocal infestation, facilitated by delayed diagnosis. Age-related hair thinning is characterized primarily by a reduction in follicular density—that is, fewer hairs per unit area—rather than a reduction in individual hair shaft diameter. This reduced density, resulting in greater interfollicular spacing, could contribute by creating a scalp environment structurally closer to that of the pubic or axillary regions and more permissive for parasite attachment and movement, although this hypothesis remains speculative [14,15]. Reduced ability to self-inspect the scalp and a lower index of clinical suspicion for pediculosis pubis in elderly patients may also contribute to delayed diagnosis.

Beyond the rarity of scalp involvement itself, infestation in a 91-year-old patient is exceptional within an already uncommon presentation. The 11 previous reports summarized in Table 1 include 19 patients with documented scalp involvement. Most publications describe isolated cases or small family clusters, with scalp involvement occurring either alone or in association with other hair-bearing sites, particularly the eyelashes and eyebrows, but also the neck, back, and pubic region. Among older adults, published cases remain particularly scarce. Individually reported cases in patients aged 65 years or older include a 92-year-old woman [16] and a 77-year-old woman in whom scalp infestation preceded eyelid involvement [10]. In addition, Eto et al. reported an institutional outbreak involving nine elderly bedridden women (mean age, 90.8 years; range, 71–98 years), seven of whom had scalp involvement, either isolated or associated with eyebrow and/or eyelash infestation [17]. Our 91-year-old patient therefore represents one of the oldest individually reported cases of scalp phthiriasis and is notable for the simultaneous involvement of the scalp, eyelashes, eyebrows, and pubic region (Table 1).

**Table 1:** Published cases of scalp infestation by *Pthirus pubis*.

**Reference**	**Year / Country**	**Age, sex**	**Sites involved**	**Treatment / Outcome**
Signore et al. [6]	1989 / USA	25 y, F	Scalp	1% lindane shampoo; eradicated at 1-week follow-up (3 children, presumed source, also treated successfully)
Silburt & Parsons [9]	1990 / Canada	6 weeks, M	Scalp	Infant treated with RID (pyrethrins/piperonyl butoxide), retreated at 1 week; mother treated concurrently with lindane; infant's eruption cleared, no recurrence at 1-year follow-up
Singh et al. [7]	1990 / India	4 y, M 8 y, M 10 y, M	Scalp	Eyelash/eyebrow involvement: eradicated with 20% fluorescein dye; scalp: benzyl benzoate + DDT (10:1) lotion, applied 3 consecutive days, successful in all 3 siblings
Klaus et al. [8]	1994 / Israel	3.5 y, F	Scalp, neck, eyelashes, back, pubis	1% permethrin cream rinse (body/scalp, 10 min), repeated at day 10; eyelashes treated with permethrin solution via cotton swab; no viable lice/eggs at 2 weeks, no side effects
Ikeda et al. [2]	2003 / Japan	4 y, F	Scalp, eyelashes	Eyelashes: manual removal + cutting lashes at base (no phenothrin, ocular irritant); scalp: phenothrin shampoo every 3 days × 4; cleared at 2 and 4 weeks
Akhoundi et al. [16]	2016 / France	92 y, F	Scalp	Malathion 0.5% + vinegar rinse (to remove nits); complete disappearance of lice at 24 h
Eto et al. [17]	2017 / Japan	92 y, F (index case); outbreak involving 8 additional elderly women (71–98 y)	Scalp involvement in 7/9 patients; variable eyebrow/eyelash involvement; pubic-only infestation in 2/9	Hair cropped + 0.4% permethrin shampoo, then Vaseline to suffocate lice + diluted vinegar to remove nits; checked every 2 weeks, considered eliminated after 2 months (all 9 patients)
Veraldi et al. [4]	2018 / Italy	37 y, F	Scalp	Foam with 0.165% pyrethrins + 1.65% piperonyl butoxide (1 application/day × 2 days, repeated at day 10); negative at 10-month follow-up
Zhang et al. [10]	2023 / China	77 y, F	Scalp, followed by eyelids	5% permethrin lotion × 3 weeks (scalp), resolved; subsequent eyelid involvement treated with 1% permethrin lotion + eyelash trimming, cleared
Nie et al. [12]	2023 / China	52 y, F	Scalp	Fine-tooth head-lice comb + sulfur soap wash (scalp)
Zhu et al. [13]	2023 / China	4 months, F	Scalp	Hair shaving + topical Stemona root tincture; parents reported cure at telephone follow-up (no in-person re-examination)
**This case**	**2026 / France**	**91** y**, F**	**Scalp, eyelashes, eyebrows, pubis**	**Topical permethrin 1% + oral ivermectin (200 µg/kg, D1 and D8); complete clinical and parasitological resolution, no recurrence**

NR: not reported.

DDT: dichlorodiphenyltrichloroethane

Several factors may contribute to delayed diagnosis. First, chronic scalp pruritus in elderly patients is more frequently attributed to seborrheic dermatitis, xerosis, eczema, or pediculosis capitis. Second, clinicians may not initially consider pubic lice in elderly individuals because pediculosis pubis is generally regarded as a sexually transmitted infestation occurring predominantly in younger, sexually active adults. However, non-sexual transmission may also occur through close physical contact or indirectly through contaminated fomites, including clothing, towels, or bedding [17]. In our patient, no sexual exposure was reported and no identifiable source of infestation was found. A non-sexual route of acquisition may therefore be considered, although this remains hypothetical and the precise mode of transmission could not be established.

Our patient illustrates both diagnostic pitfalls. She underwent empirical antifungal treatment for two months before careful examination of the scalp revealed numerous lice and nits. The simultaneous involvement of the eyelashes, eyebrows, and pubic region strongly supported the diagnosis and prompted parasitological confirmation. A comparable diagnostic sequence — scalp infestation preceding eyelid involvement — has been reported in a 77-year-old woman, underscoring the value of systematically examining all hair-bearing areas once pediculosis pubis is suspected [10].

Treatment of pediculosis pubis relies mainly on topical pediculicides such as permethrin, while oral ivermectin may be considered in extensive, refractory, or multifocal infestations [1,18,19]. Repeated treatment is often necessary because neither topical insecticides nor ivermectin possess ovicidal activity [18,19].

This case emphasizes the importance of performing a complete examination of all hair-bearing areas in any patient presenting with unexplained scalp pruritus, regardless of age. Early recognition allows prompt treatment and avoids unnecessary investigations or inappropriate therapies.

## Conclusion

Scalp infestation by *Pthirus pubis* is an unusual presentation that may lead to delayed diagnosis, particularly in elderly patients. Careful clinical examination and parasitological confirmation remain essential for accurate diagnosis. Clinicians should consider pubic lice in the differential diagnosis of chronic scalp pruritus, especially when other hair-bearing areas are simultaneously involved.

## CRediT authorship contribution statement

**Floriane Etheve:** Writing – review & editing, Writing – original draft, Visualization, Data curation. **Estelle Perraud:** Investigation, Methodology, Validation, Writing – review & editing. **Damien Boutin:** Writing – review & editing, Supervision, Investigation.

## Ethics declaration

Written informed consent to take part in the study and to publish the article has been obtained from all participants or their legal representatives. The privacy rights of participants have been observed.

This study was performed in compliance with relevant laws, regulatory frameworks and guidelines where the research took place. Ethics committee approval was not required under relevant laws and institutional guidelines. According to our institutional policy, ethics committee approval was not required for this single anonymized case report.

This research follows the CARE guidelines and the CARE checklist.

## Funding

This research did not receive any specific grant from funding agencies in the public, commercial, or not-for-profit sectors.

## Declaration of Competing Interest

The authors declare that they have no known competing financial interests or personal relationships that could have appeared to influence the work reported in this paper.
